# Negative density-dependent dispersal in the American black bear (*Ursus americanus*) revealed by noninvasive sampling and genotyping

**DOI:** 10.1002/ece3.207

**Published:** 2012-03

**Authors:** Justin Roy, Glenn Yannic, Steeve D Côté, Louis Bernatchez

**Affiliations:** 1 Département de Biologie, Université Laval,Quebec, QC, G1V 0A6, Canada; 2 Institut de Biologie Intégrative et des Systèmes (IBIS), Université Laval,Quebec, QC, G1V 0A6, Canada; 3 Centre d’Études Nordiques, Université Laval,Quebec, QC, G1V 0A6, Canada

**Keywords:** Black bear, dispersal, inbreeding avoidance, philopatry, population density, *Ursus americanus*

## Abstract

Although the dispersal of animals is influenced by a variety of factors, few studies have used a condition-dependent approach to assess it. The mechanisms underlying dispersal are thus poorly known in many species, especially in large mammals. We used 10 microsatellite loci to examine population density effects on sex-specific dispersal behavior in the American black bear, *Ursus americanus*. We tested whether dispersal increases with population density in both sexes. Fine-scale genetic structure was investigated in each of four sampling areas using Mantel tests and spatial autocorrelation analyses. Our results revealed male-biased dispersal pattern in low-density areas. As population density increased, females appeared to exhibit philopatry at smaller scales. Fine-scale genetic structure for males at higher densities may indicate reduced dispersal distances and delayed dispersal by subadults.

## Introduction

Natal dispersal, defined as the movement of an individual from its birth site to the place where it might reproduce (Howard 1960), has been hypothesized to play a major role in population regulation (Hestbeck 1982), metapopulation and source–sink dynamics (Dias 1996), as well as influencing the population genetics of species (Bohonak 1999). Natal dispersal's complement, that is, natal philopatry, is also of great interest in behavioral ecology given its potential implication in the evolution of kin selection (Waser and Jones 1983). In fact, it is difficult to imagine any ecological or evolutionary process that is not affected by dispersal (Dieckmann et al. 1999). Four key factors are currently recognized to affect the evolution of dispersal (reviewed in Lawson Handley and Perrin 2007): inbreeding avoidance (Pusey 1987), local resource competition (Clark 1978), local mate competition (Dobson 1982), and cooperative behavior among kin (Perrin and Lehmann 2001). Although much effort has been devoted to this question, considerable controversy persists about the relative importance of each factor in shaping patterns of dispersal (Lambin et al. 2001). As a result, dispersal remains one of the most studied, yet least understood life-history traits (Clobert et al. 2001). Part of the challenge stems from the complex interactions that might exist among the above factors (Gandon and Michalakis 2001), whose importance could also vary according to the species (Pusey and Wolf 1996), and the spatiotemporal scale investigated (Ronce et al. 2001).

A milestone in the study of dispersal has been provided by Greenwood (1980), who reported male-biased dispersal and female philopatry in most mammalian species. The majority of the subsequent dispersal studies have corroborated these conclusions (for a review, see Table 1 in Lawson Handley and Perrin 2007). Sex-biased dispersal has therefore important consequences for the genetic makeup of populations (Clobert et al. 2001). Most studies that investigated dispersal, however, did not go beyond reporting the sex-bias pattern and as such, it is often difficult to draw clear conclusions regarding the factors influencing dispersal behavior. A complementary approach consists in studying both environmental and internal factors underlying dispersal of animals (termed condition-dependent dispersal, Ims and Hjermann 2001). Examples of environmental factors typically include habitat and food quality, population density, and social structure, whereas internal factors typically refer to fat reserves, body size, and competitive ability of individuals (Ims and Hjermann 2001). Studies aim at understanding how variation in one (or more) of these factors might affect dispersal behavior, and have the potential to provide valuable insights into the costs and benefits of dispersal for each sex (Bowler and Benton 2005).

**Table 1:** Polymerase chain reaction (PCR) conditions for the 10 microsatellite loci used in the study of black bears fine-scale genetic structure, along with postamplification mix sets when ran on a 3100 ABI sequencer. *T°* C indicates the optimal annealing temperature.

Locus	Fluorescent dye labeling	*T*°C	MgCl_2_ (mM)	Primers (μM)	*Taq* (units)	Post-PCR mix
G1D	FAM	56.0	1.9	0.2	1.0	mix 1
G10H	FAM	59.0	1.9	0.2	1.0	mix 1
G10L	NED	56.0	1.9	0.3	2.0	mix 2
G10M	HEX	62.0	1.6	0.3	0.6	mix 4
G10P	FAM	58.0	1.5	0.4	1.3	mix 3
MU09	NED	60.0	1.0	0.3	1.5	mix 1
MU10	HEX	59.0	1.2	0.3	2.5	mix 4
MU15	FAM	56.0	0.8	0.3	1.6	mix 1
MU23	HEX	55.0	1.2	0.5	2.0	mix 3
MU50	HEX	55.0	2.0	0.5	1.6	mix 2

Density-dependent dispersal has been found to occur in natural populations (Ims and Hjermann 2001), where the dispersal rate may either increase (positive density dependence) or decrease (negative density dependence) with population density. While the existence of density-dependent dispersal is well documented in invertebrates (e.g., Fonseca and Hart 1996), few studies have explicitly focused on this topic in birds and mammals (reviewed in Matthysen 2005). Despite considerable theoretical interest in the form of the density-dependent dispersal function in population regulation (Sæther et al. 1999; Travis et al. 1999), empirical evidence of density dependence, in medium- and large-sized mammals is scarce (Matthysen 2005; Støen et al. 2006; Loe et al. 2009). Clearly, a complete picture of the factors influencing dispersal requires additional empirical investigations.

The American black bear (*Ursus americanus*) is a generalist, opportunist, and solitary species distributed over a wide range of population densities in North America. Although it has often been reported that most subadult males disperse from their natal area whereas most females settle in or adjacent to it (Rogers 1987b; Schwartz and Franzmann 1992; Costello 2010), some observations suggest that dispersal may be a more complex process, influenced by population density. Indeed, interpopulation comparisons revealed variation in the age of sexual maturity and dispersal among males exposed to different density regimes (Lindzey and Meslow 1977; Rogers 1987a). Coupling microsatellite DNA and spatial data, Costello et al. (2008) notably showed that males in lower density areas dispersed less often or to shorter distances than males in higher density areas. Schenk et al. (1998) reported no evidence for female philopatry in a high-density population in Ontario, Canada, and speculated that the general population structure described elsewhere for black bears may occur only under certain density conditions. Taken together, these observations illustrate the need to assess the influence of population density on the dispersal decision in black bears, and might provide informative data on its potential effects on the cost-to-benefit ratio of dispersal in large-sized mammals in general. Furthermore, during the past two decades, many American black bear populations have increased numerically and expanded geographically (Williamson 2002; Garshelis and Hristienko 2006; but see Beston 2011) that may have affected dispersal behavior. While sex biases in dispersal can be estimated by methods that rely on field observations of individual movements (e.g., mark capture, radio tracking, etc.), alternative methods based upon genetic data are often more applicable for species that are difficult to observe, capture, and mark (for a methodological review, see Broquet and Petit 2009; and for an example, see Harris et al. 2009).

Our objective was to examine using noninvasive sampling and microsatellite genotyping the effect of population density on sex-specific dispersal behavior in the American black bear. We tested the hypothesis of positive density-dependent dispersal in black bears, as suggested for most mammal species (Matthysen 2005), namely an increased dispersal rate for both sexes as population density increases. Accounting for the typical male-biased dispersal pattern in mammals (Greenwood 1980), we predicted: (1) a fine-scale genetic structure for females, but not for males, at low population densities; and (2) no fine-scale genetic structure for both sexes at higher densities.

## Materials and Methods

### Study area

**Figure 1:**
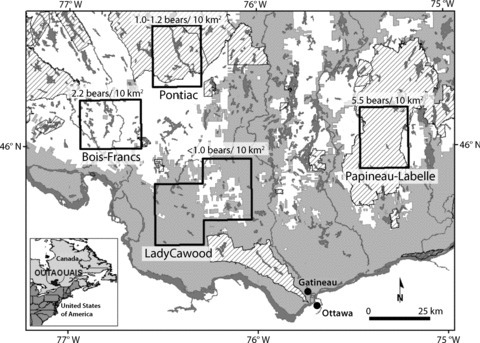
The study area located in Outaouais, Québec, Canada (approximately 46°N, 76°W), was divided into four sampling areas (dark polygons): Pontiac, LadyCawood, Bois-Francs, and Papineau-Labelle. White and light grey areas denote public and private properties, respectively. Dashed areas indicate delegate management territories.

The study area was located in Outaouais (approximately 46°N, 76°W; Fig. 1), in southwestern Québec, Canada. It is dominated by mature deciduous forests of sugar maple (*Acer saccharum*), red maple (*Acer rubrum*), yellow birch (*Betula alleghaniensis*), and American beech (*Fagus grandifolia*). Four sampling areas were distributed throughout the region in areas with different densities of bears (Fig. 1). The first sampling area (500 km^2^) is part of the Pontiac Zone (hereafter named Pontiac), for which an estimate of 1.0–1.2 bears/10 km^2^ was observed. The second sampling area (1000 km^2^) was located in the Lady Smith-Cawood area (hereafter named LadyCawood), for which the bear density level was low (<1 bear/10 km^2^). The third sampling area (500 km^2^), referred to as Bois-Francs, has been characterized by a relatively high bear density, namely 2.2 bears/10 km^2^. The fourth sampling area (500 km^2^) is part of the Papineau-Labelle Wildlife Reserve (hereafter named Papineau-Labelle), known for its high density of bears (5.5 bears/10 km^2^). With the exception of Papineau-Labelle (Jolicoeur and Lemieux 1990), all density estimates were obtained from a genetic capture-mark-recapture (CMR) study conducted in 2005 (Roy et al. 2007). Hereafter, we considered as high-density area with ≥2.2 bears/10 km^2^ (i.e., Papineau-Labelle and Bois-Francs) and low-density area with ≤1.2 bears/10 km^2^ (i.e., LadyCawood and Pontiac). All sampling areas were distributed in a relatively large homogeneous landscape (Goudreault and Toussaint 2005), thus excluding differences in habitat quality as the main factor explaining the fine-scale genetic patterns obtained in this study.

### Samples collection

Sampling was conducted in summer 2005 between 4 July and 4 August. Samples were obtained from barbed wire hair traps. In order to provide adequate scaling for the study of fine-scale genetic structure, each sampling area was divided into 20 (40 for LadyCawood) 5 × 5-km cells. One station was built within each cell, except for two cells (four for LadyCawood) per area that contained five stations. The average distance between each of the 140 stations and the nearest one was 3.22 km (SD = 0.90 km), and Universal Transverse Mercator (UTM) geographic coordinates were recorded for all stations using Global Positioning System (GPS). We visited each station on a weekly basis, removed hair samples, sterilized barbed wire, and refreshed the food lure as necessary. All hairs collected on the same side of the barbed wire defined a sample, and all samples were preserved dry at room temperature into individual paper envelops until DNA extraction. A total of 411 hair samples were collected at 249 stations, that is, 175 hair samples at 72 stations for Pontiac, 90 at 56 stations for LadyCawood, 107 at 66 stations for Bois-Francs, and 89 at 55 stations for Papineau-Labelle (Table 3).

**Table 2:** Allelic dropout (ADO) and false allele (FA) rates for five DNA content-based categories, as determined by a pilot study conducted on 18 hair samples at seven microsatellite loci (G1D, G10H, G10L, MU09, MU15, MU23, and MU50). The positive PCR rate as well as the total number of independent PCRs (no. of PCRs) needed to obtain single-locus genotypes at the 99% confidence level are also reported.

Category	Positive PCR rate	ADO	FA	No. of PCRs	*N cat.*
1–2 hairs	94.0% (378/402)	6.3% (18/288)	5.8% (22/378)	2	9
3–4 hairs	97.3% (395/406)	0.7% (2/299)	1.3% (5/395)	1	44
5–6 hairs	99.1% (347/350)	0.4% (1/277)	0.0% (0/347)	1	23
7–9 hairs	98.7% (380/385)	0.0% (0/298)	0.3% (1/380)	1	98
10+ hairs	98.3% (399/406)	0.0% (0/302)	0.0% (0/399)	1	78

Numbers in parentheses refer to the number of observed cases on the total number of potential cases.

*N cat*, number of samples of each category included in the final dataset.

**Table 3:** Sampling characteristics of the four study areas used, along with the sex denotation of the 141 individuals genetically identified (No of stations = total number of stations visited by a bear during the whole study period; No. of samples = total number of samples for which DNA analyses were conducted; No. of females; and No. of males = number of unique genotypes obtained for females and males, respectively).

Sampling area	Density of bears/10 km^2^	No. of stations	No. of samples	No. of females	No. of males
Pontiac	1.0–1.2	72	125	25	10
LadyCawood	<1.0	56	90	15	15
Bois-Francs	2.2	66	107	23	22
Papineau-Labelle	5.5	55	89	17	14
Total		249	411	80	61

**Table 4:** Summary of the genetic variation characteristics of the 10 microsatellite loci used, obtained from the whole sample of 141 individuals.

Locus	No. of alleles	Allelic range	*P_(ID)Sib_*	*H*_O_	*H*_E_	*F*_IS_
G1D	9	(174–190)	0.4027	0.716	0.747	0.041
G10H	20	(230–270)	0.2929	0.936	0.923	−0.014
G10L	13	(133–165)	0.3216	0.836	0.874	0.044
G10M	9	(192–208)	0.3466	0.841	0.834	−0.007
G10P	11	(163–185)	0.3632	0.794	0.807	0.016
MU09	10	(187–209)	0.3631	0.780	0.809	0.035
MU10	10	(116–138)	0.3232	0.879	0.872	−0.008
MU15	9	(126–142)	0.3594	0.794	0.813	0.023
MU23	11	(153–175)	0.3248	0.851	0.869	0.021*
MU50	13	(111–141)	0.3258	0.865	0.868	0.003
Overall	11.5	(111–270)	2.131.10^−5^	0.829	0.842	0.015

* Significant values (α= 0.05) based on 10,000 permutations.

*P_(ID)Sib_*, probability of identity among siblings; *H*_O_, observed heterozygosity; *H*_E_, expected heterozygosity; *F*_IS_, inbreeding coefficient).

### Pilot study

In order to minimize genotyping errors, we conducted a pilot study to determine allelic dropouts (ADO: one allele of a heterozygous individual is not amplified during a positive polymerase chain reaction [PCR]), and false alleles (FA: PCR-generated allele as a result of a slippage artefact during the first cycles of the reaction) (Taberlet et al. 1996) associated with five different DNA content-based categories. We then used the ADO estimates to design an optimal genotyping protocol yielding reliable single-locus genotypes at the 99% certainty level, as previously described in Morin et al. (2001). The selected categories were 1–2 hairs (category 1), 3–4 hairs (category 2), 5–6 hairs (category 3), 7–9 hairs (category 4), and 10+ hairs (category 5). For each category, DNA was extracted for a total of 18 different hair samples taken randomly within all samples, and amplified seven times (following Taberlet et al. 1996) to a variable number of markers among seven of the 10 loci used in this study (i.e., G1D, G10H, G10L, MU09, MU15, MU23, and MU50). The selected loci were chosen to cover the whole range of allelic sizes found in our study (see Table 4), which was shown to influence the locus-specific genotyping error rate (Broquet et al. 2007). A given sample was used to test all five categories at a particular locus, such that a consensus genotype could be derived easily from the comparison of the different PCR products over all categories. We then calculated, for each category, over all loci error rates according to equation 2 for ADO and equation 4 for FA of Broquet and Petit (2004), as well as the frequency of positive PCR amplification.

### DNA extraction and microsatellite analysis

DNA extraction from hair samples was carried out using the DNeasy Protocol for Animal Tissues (Qiagen Inc., Valencia, CA) with minor modifications. Up to 10 guard hair roots as available were cut off and placed into 1.5-mL centrifuge tubes containing 180 µL of ATL buffer. Twenty microlitres of proteinase K (20 mg/mL) and 30 µL of DTT (100 mg/mL) were added to each tube before incubating at 37°C overnight. The remaining steps of the DNA extraction followed exactly those described in the DNeasy Tissue Kit Handbook (Qiagen Inc.), except for the last procedure in which the elution volumes were adjusted for each sample according to the hair category to which it belonged: 60 µL (category 1), 70 µL (category 2), 75 µL (category 3), 80 µL (category 4), or 100 µL (category 5), as in the pilot study.

All samples were genotyped at 10 microsatellite loci using sets of primers developed from black and brown bear samples: G1D, G10L (Paetkau and Strobeck 1994); G10H, G10M, G10P (Paetkau et al. 1995); and UarMU09, UarMU10, UarMU15, UarMU23, UarMU50 (Taberlet et al. 1997). For each hair sample, all loci were initially PCR amplified twice as suggested by the pilot study (see results), with each marker being amplified in a single reaction using a Biometra® thermocycler (Goettingen, Germany). All reactions were performed in a 15-µL reaction volume containing 1.5 µL of 10× reaction buffer (100-mM Tris-HCl pH 9.0, 1% Triton X-100, 500-mM KCl), 0.4 µL of dNTP (2.5 mM each), 2.0 µL of BSA (1.0 mg/mL), and 1.5 µL of template DNA. The concentrations of MgCl_2_, primers, and *Taq* polymerase were optimized for each locus, as well as the annealing temperature during the PCR cycles (Table 1). The amplifications included an initial denaturation step of 2 min at 94°C, followed by 35 cycles of 45 sec at 94°C, 45 sec at the selected annealing temperature, and 45 sec at 72°C, completed by a 5-min final elongation step at 72°C. PCR products were pooled in four kits of loci (Table 1), ran on a 3100 ABI sequencer and analyzed with genescan 3.7.1 and genotyper 3.7 softwares (Applied Biosystems Inc., Foster City, CA). Additional amplifications to correct for negative PCRs and ambiguous results were performed as necessary.

### Sexing protocol

Sex identification was carried out twice for each sample using the protocol of Yamamoto et al. (2002). The reaction was performed in a 20-µL reaction volume containing 2.0 µL of 10× reaction buffer (100 mM Tris-HCl pH 9.0, 1% Triton X-100, 500-mM KCl, 1.0-mM MgCl_2_), 1.8 µL of dNTP (2.5-mM each), 1.8 µL of each SE47 and SE48 primers (10 µM each), 2.5 µL of BSA (1.0 mg/mL), 1.2 U of *Taq* polymerase (1.0 U/µL), and 5.0 µL of template DNA. The amplification consisted of an initial denaturation step of 9 min at 95°C, followed by 70 cycles of 30 sec at 94°C, 30 sec at 60°C, and 1 min at 72°C, completed by a 5-min final elongation step at 72°C. Ten microlitres of each PCR product were run on a 2.5% agarose gel and negative controls were used during the whole process.

### Individual identification

The program identity 1.0 (Wagner and Sefc 1999) was used to identify all potential recapture cases of an individual from the whole dataset of 10-locus genotypes. A complete list of unique genotypes was derived manually. We performed two tests implemented in the software dropout (McKelvey and Schwartz 2005) to identify potential genotyping errors. The “bimodal test” reported the minimum number of loci different between each sample and its most similar sample, whereas the “difference in capture history test” targeted those loci likely producing the errors. dropout was also used to calculate the conservative probabilities of identity among siblings (*P*_ID(sib)_, Waits et al. 2001) for each locus and over all loci.

### Standard genetic analyses

fstat 2.9.3 (Goudet 1995) provided the following locus-specific information when all individuals were considered in the analyses: number and range of alleles, observed and expected heterozygosities (Nei 1978), as well as inbreeding coefficients (*F*_IS_, Weir and Cockerham 1984) and their statistical significance based on 10,000 permutations. We used the program genepop 3.4 (Raymond and Rousset 1995) to test for departure from Hardy–Weinberg equilibrium on a per locus basis and for linkage disequilibrium between pairs of loci, in each sampling area. Markov chain parameters for all tests were set at 10,000 dememorizations, 1000 batches, and 10,000 iterations.

### Individual-based genetic structure

*F*_ST_ coefficients and their 95% confidence intervals (estimated via bootstrapping over loci) were computed between pairs of sampling areas with fstat. To test differentiation among populations, we used the exact *G*-test on allelic frequencies (Goudet et al. 1996) as implemented in fstat (10,000 randomizations). Sampling areas showing nonsignificant or weak genetic differentiation were then combined into a single genetic group for the calculation of reference allele frequencies.

Spatial genetic structure at the individual level was examined in each sampling area using Mantel tests (Mantel 1967) and multilocus spatial autocorrelation analyses (Smouse and Peakall 1999). The comparisons involved in both types of methods were for all individuals, female–female pairs and male–male pairs. The null hypothesis of no spatial genetic structure was tested against the alternative hypothesis of fine-scale genetic structure expected under philopatry (females) or restricted dispersal (all individuals, males). Pairwise relatedness coefficient between individuals (*r_ij_*, following Queller and Goodnight 1989) was first computed and linearly regressed on the natural logarithm of pairwise geographic distance. The coefficient of determination (*R*^2^) was calculated for each comparison using the program SpaGedi 1.2g (Hardy and Vekemans 2002). Elements of the individual locations matrix were permuted 20,000 times (cf. Mantel test) to test for the significance of the observed regression slope (α= 0.05). The spatial coordinates of an individual were defined as the arithmetic mean of its total genetic-capture locations (when captured more than once). Ln-transformation of spatial distances was applied to exclude from the analyses potential mother–offspring pairs with identical spatial coordinates that reduces the probability to detect an artefactual substructure created by young animals that have yet to disperse.

Because we were also interested in obtaining a detailed picture of how relatedness between two individuals changed with the distance separating their mean locations, we further investigated the fine-scale genetic structure using a spatial genetic autocorrelation technique implemented in the software genalex 6 (Peakall and Smouse 2006). This technique has been described in detail in previous studies (e.g., Peakall et al. 2003; Double et al. 2005). In order to increase statistical testing power for each distance class, we took advantage of the *Multiple Pops* option allowing the autocorrelation coefficient (*r*) to be calculated across multiple sets of individuals (*rc*). Based upon the natural dichotomy of the bear population density levels and the highly similar genetic patterns among some sampling areas as revealed by the Mantel tests (see results), we carried out spatial genetic autocorrelation analyses across (1) Pontiac and LadyCawood sampling areas, and (2) Bois-Francs and Papineau-Labelle sampling areas. These associations are hereafter referred to as low-density and high-density areas, respectively. For each type of comparison, we first defined distance classes as a trade-off between the spatial resolution and the number of pairs in each class: 1 km, 4 km, 7 km, 10 km, 15 km, and 30 km. The first distance class (1 km) contained only comparisons between individuals with identical spatial coordinates. To avoid the bias described above, our interest here was exclusively in the detection of positive spatial autocorrelation in the second distance class (4 km), as expected under philopatry (females) or dispersal across short distances (all individuals, males). Because we also wanted to assess the influence of the second distance class size chosen on the interpretation of the results, we also performed the same analyses but modifying only the second distance class as follows: every 1 km from 4 to 10 km, successively. Results are presented as correlograms (plots of *rc* as a function of distance), with 95% confidence interval about *rc* estimated by 1000 bootstraps. Positive spatial genetic structure was declared when the probability *P* to achieve a value greater than or equal to the observed *rc* was less than 0.05, as determined through 10,000 random permutations of the individual genotypes among the geographic locations

## Results

### Pilot study

Positive PCR frequency ranged from 94.0% to 99.1% for the five DNA content-based categories (Table 2). Both ADO and FA rates showed a decreasing tendency as the number of guard hairs used in the DNA extraction increased. Allelic dropouts varied between 0% and 6.3%, whereas FA ranged from 0% to 5.8% (Table 2). Assuming a uniform DNA quality over all categories, these results indicate that DNA quantity influenced the genotyping error rates in our study. In Table 2, we present the number of repetitions required for each category to obtain a reliable single-locus genotype at a 99% confidence level. For the category 1, at least two repetitions were required to achieve a reasonable level of genotyping accuracy, while a single amplification was enough for the four other categories.

### Individual identification

The multilocus genotype of at least one individual was successfully identified at 226 of the 249 stations, among which a second genotype was further detected at 17 stations (Table 3). Based upon these 243 genotypes, identity 1.0 (Wagner and Sefc 1999) identified 141 unique individuals. The “bimodal test” conducted in dropout (McKelvey and Schwartz 2005) revealed a fiveto eight loci differentiation between each pair of individuals, whereas the “difference in capture history test” did not identify any new individual following permutations of the loci (*L_base*= 6 loci). Genotyping errors were thus reasonably minimal in our dataset. The overall probability of identity among siblings (*P*_ID(sib)_) was 2.131 × 10^–5^ (range 0.293–0.403 per locus, Table 4), thereby confirming sufficient power to discriminate between individuals in the study area. A total of 80 females and 61 males were genetically identified, and the sex ratio of the samples was unbiased in all sampling areas except for Pontiac (Table 3).

### Standard genetic analyses

The total number of alleles per locus varied between nine and 20, with an average of 11.5 (Table 4). Observed heterozygosity values (range 0.72–0.94 per locus) were similar in most cases to those expected under random union of gametes (range 0.75–0.92 per locus), and *F*_IS_ values were all nonsignificant (*P* > 0.05) except for locus MU23 (*F*_IS_= 0.021, *P*= 0.012). Given the weak signal of inbreeding at this locus and the fact that nine of 10 loci did suggest random mating in the whole sample, we did not reject that locus from the dataset. Global *F*_IS_ value was 0.015, also not significant (*P*= 0.45). When tested for each locus in each sampling area, significant departure from Hardy–Weinberg equilibrium was found at only two loci (G1D in Papineau-Labelle, MU23 in Pontiac). Given this proportion is expected to occur by chance alone (2/40 = 0.05), we concluded that the assumption of Hardy–Weinberg equilibrium was respected in each sampling area. Fifteen pairs of loci deviated significantly (α= 0.05) from linkage equilibrium, a number slightly over the one expected by chance alone (15/180 = 0.083). Because these deviations did not involve twice the same pair of loci and might result from the existence of more than one genetic group in the whole study area (see individual-based genetic structure), all loci were assumed to be statistically independent and were retained for genetic structure analyses.

### Individual-based genetic structure

Pairwise *F*_ST_ coefficients between sampling areas were all significantly different from zero (all *G*-tests, *P* < 0.05). The main divergence was observed between Papineau-Labelle and the three other areas (range: 0.029–0.032). In contrast, the *F*_ST_ values between the other three sampling areas were very weak, averaging 0.005 (range: 0.004–0.009). We thus concluded that there were two major genetic groups in the dataset, namely Papineau-Labelle (*n*= 31 individuals) and the one composed of remaining individuals (*n*= 110) located in Pontiac, LadyCawood, and Bois-Francs (see Fig. 1 for locations). Therefore, for Mantel tests, interindividual comparisons used, independently, reference allele frequencies obtained (1) from all individuals captured in Papineau-Labelle sampling site and (2) from all individuals within each sample site of Pontiac, LadyCawood, and Bois-Francs, respectively.

Mantel test revealed a significant negative relationship between *r_ij_* values and ln-distance among pairs of females in Pontiac, a sampling site located in an area of low bear density, and was nearly significant for the same comparison in LadyCawood (*P*= 0.048 and 0.082, respectively, Table 5), located also in a low density area. For both of these sampling areas, comparisons involving either all individuals or males only were nonsignificant (Table 5). In contrast, for both sampling sites located in areas of relatively high bear density (i.e., Bois-Francs and Papineau-Labelle), a significant negative relationship was obtained for male–male dyads but not for female–female dyads (Table 5). When including all individuals, the relationship was nearly significant for Bois-Francs (*P*= 0.067, Table 5) and significant for Papineau-Labelle (*P*= 0.015, Table 5).

**Table 5:** Results of the linear regressions between interindividual pairwise relationship coefficient (*r_ij_*) and the natural logarithm of geographic distance separating two individuals. For all sampling areas, comparisons are shown for all individuals, females only, and males only. The coefficient of determination (*R*^2^), the probability (*P*) to obtain a regression slope lower than the one observed, and the number of pairwise comparisons (*n*) are reported.

	All individuals			Females only			Males only		
Sampling area	*R*^2^	*P*	*n*	*R*^2^	*P*	*n*	*R*^2^	*P*	*n*
Pontiac	0.000	0.306	577	0.013	0.048*	291	0.019	0.775	41
LadyCawood	0.000	0.530	322	0.023	0.082	81	0.039	0.962	77
Bois-Francs	0.003	0.067	960	0.004	0.148	245	0.013	0.039*	219
Papineau-Labelle	0.014	0.015*	454	0.000	0.442	133	0.048	0.024*	90

* Significant values (*P* < 0.05) based on 20,000 permutations.

**Figure 2:**
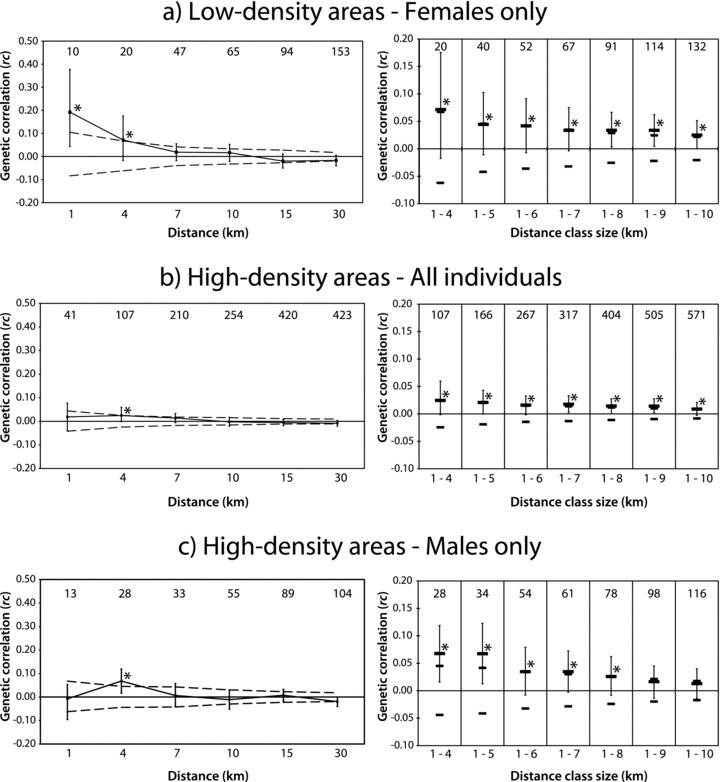
Left panels: correlogram plots of the genetic correlation coefficient (*rc*) across low-density and high-density areas of black bear as a function of geographic distance. For simplicity, only correlograms depicting significantly positive *rc* values (coded by asterisks) within the 4-km distance class are shown. All individuals with identical spatial coordinates fall within the 1-km distance class. (a) Low-density areas – females only (*n*= 40); (b) high-density areas – all individuals (*n*= 76); (c) high-density areas – males only (*n*= 36). The 95% confidence interval for the null hypothesis of a random distribution of genotypes (dashed lines) and the bootstrapped 95% confidence error bars are also shown. The number of pairwise comparisons within each distance class is presented above the plotted values. Right panels: graphs showing the influence of different second class sizes on the spatial autocorrelation analyses for cases considered in the left panels. Only the second distance class is shown, for increasing distance class sizes from 4 to 10 km. The thicker line denotes the genetic correlation coefficient (*rc*), and the thinner lines indicate lower and upper bounds of the 95% confidence interval for the null hypothesis of a random distribution of genotypes. Bootstrapped 95% confidence error bars are also shown. The number of pairwise comparisons within each distance class size is presented above the plotted values. Asterisks denote significantly positive *rc* values (*P* < 0.05).

Spatial autocorrelation analysis of females located in the low-density areas revealed significantly positive *rc* value in the 4-km distance class (*P*= 0.018, Fig. 2a), which was not the case when either all individuals or only males were considered in the analyses (*P*= 0.113 and 0.782, respectively, correlograms not shown). In contrast, spatial autocorrelation analyses of all individuals and male genotypes located in high density areas revealed significantly positive *rc* value within the 4-km distance class (*P*= 0.028 and 0.003, respectively, Fig. 2b and c), whereas females did not depart from a random distribution of genotypes for the same distance class (*P*= 0.683, correlogram not shown). Fig. 2 also indicate that the interpretation of the current results is not dependent on the second distance class size chosen, as suggested by the positive *rc* values that declined but remained significant beyond the distance class size of 8 km.

## Discussion

The main objective of the study was to examine the effect of population density on sex-specific dispersal behavior in the American black bear. In the low-density areas, females in close proximity, but not males, had higher genetic similarity than expected from random. These observations are consistent with the male-biased dispersal pattern and female-biased philopatry commonly reported in mammals (Greenwood 1980; Lawson Handley and Perrin 2007). Genetic evidence for this pattern has been documented for various species such as rodents (e.g., dusky-footed woodrat, Neotoma fuscipes, McEachern et al. 2007), bats (e.g., Bechstein's bat, *Myotis bechsteinii*, Kerth et al. 2002), or ungulates (e.g., Soay sheep, *Ovis aries*, Coltman et al. 2003), but also in Ursidae species, for example, brown bear, *Ursus arcticus* (Støen et al. 2006; Zedrosser et al. 2007), polar bear, *Ursus maritimus* (Zeyl et al. 2009), or giant panda, *Ailuropoda melanoleuca* (Zhan et al. 2007). In black bears, female-biased philopatry has mostly been suggested from field studies (e.g., Jonkel and Cowan 1971; Rogers 1987a; Costello 2010) and its genetic evidence remains rather scarce (but see Onorato et al. 2004; Costello et al. 2008). Several factors have been proposed as potential advantages that could explain female philopatry in mammals. Among them, familiarity with food resources and good denning sites in the natal area would be important for home range acquisition in black bears (Waser and Jones 1983). In bears living in the forest, knowledge of food resources is developed through experience and establishing a home range near that of the mother could be highly advantageous (Rogers 1987a). In addition, poor nutrition in adults may result in no implantation of the blastocysts, resorption of the implanted fetuses, or early death of neonates (Pelton 2003).

Our results also indicated that dispersal behavior in both sexes might be affected by an increase in population density. The nondetection of fine-scale genetic structure for females in high-density areas, despite the use of a similar sampling scheme for all areas, suggests that philopatry likely occurred at smaller scales as population density increased. Negative relationships between population density and home-range size have been shown for bears (e.g., Oli et al. 2002; Dahle and Swenson 2003). As pointed out previously (Peakall et al. 2003), sampling at intervals greater than the scale of genetic structure results in its nondetection. We believe that this scenario likely explains the nondetection of fine-scale genetic structure for females in high-density areas. In support to this assertion, Støen et al. (2005) found spatial associations of kin females both in high- and low-density areas of the brown bear distribution in Scandinavia, and they argued that female–female competition for space better explains the closer settlement of females to the natal area at higher densities (Støen et al. 2006).

Although the perspective of increased dispersal by female black bears under high-density conditions cannot be entirely ruled out on the basis of our results, we believe it is unlikely for two reasons. First, this would imply that dispersing females at high density would gain an important benefit – or at least reduce costs – compared to philopatric ones. Since population density influences age of primiparity in some mammal species (e.g., Jorgenson et al. 1993), it might be reasonable to assume that females at higher densities would disperse in order to advance primiparity. However, delayed reproduction for dispersing females compared to philopatric ones has been noted previously in black bears (Jonkel and Cowan 1971), suggesting costs for dispersing females that are perhaps associated with foraging in unfamiliar areas (Rogers 1987a). Second, increased dispersal by females at higher densities is unlikely when considering that dispersal by males is negatively affected by population density. In most vertebrate species with sex-biased dispersal, it is generally recognized that rates of dispersal of the less dispersive sex are more influenced by density than that of members of the opposite sex (Lambin et al. 2001).

The fine-scale genetic structure observed for males suggests lower dispersal rate/distance at high density than at low density. If inbreeding avoidance was the sole driver of male dispersal, it would be reasonable to assume that male dispersal would be density independent (e.g*.*, Zedrosser et al. 2007). Higher density, however, has been hypothesized to increase dispersal rates by forcing individuals to emigrate to ultimately reduce local resource competition or local mate competition (Greenwood 1980; Dobson 1982; Waser 1985). Mate competition among males has notably been suspected to be an important factor in the dispersal of black bears (Costello et al. 2008). We propose here two proximate factors that might explain the unexpected pattern of male dispersal, namely reduced dispersal distances and delayed dispersal by subadults at higher densities.

Individuals may restrict dispersal distances under high-density conditions, potentially due to increased dispersal costs during the transience and immigration stages, despite the cost associated with staying within a densely populated area. Dispersal costs for an individual include increased mortality risk in the transience stage and disadvantages during the settling period in the new environment (Gandon and Michalakis 2001; Festa–Bianchet and Côté 2008), as well as the physiological costs of movement (Sutherland et al. 2000). Evidence of reduced dispersal and greater spatial association of kin at high density has been particularly well documented in small mammals (Lambin 1994). In contrast, such evidence remains scarce in larger mammals (e.g. Støen et al. 2006), possibly reflecting the difficulty to study dispersal in such species rather than the rarity of the phenomenon. The question of dispersal costs is particularly relevant in black bears since resident adult males are known to deter immigration by subadult males (Sargeant and Ruff 2001), and females may show differential aggression against nonkin males (Rogers 1987b). These agonistic behaviors are likely to be more prevalent as density increases due to higher encounter rates between nonspecifics, making dispersal a more costly process at higher densities. As a result, the cost-to-benefit ratio of dispersal for an individual would increase, hence supporting the apparently shortest dispersal distances by males in our high-density areas. It is generally accepted in the literature that dispersal costs increase with the distance (e.g., Rousset and Gandon 2002), but our study provides an additional argument that population density should also be considered in studies of dispersal.

Dispersal by subadult males (2–4 years) may also be delayed under high-density conditions. For several species of mammals, an increase in population density means an increase in competition for space and food (Fretwell and Lucas 1970), resulting in a decrease in the per capita food abundance. As a consequence, maternal expenditure and growth rate of offspring are generally adversely affected under more limiting environmental conditions (Therrien et al. 2007). In the Scandinavian brown bear, *Ursus arctos*, it has been demonstrated that the size of adult females decreased with increasing population density (Zedrosser et al. 2006), as did both size and mass of yearlings (Dahle et al. 2006). For males, a decrease in growth rate in early age could extend the physical growth period and delay sexual maturity as well as dispersal. We thus hypothesize that successful male black bear dispersers might be older in high-density areas than at lower densities. Interpopulation differences in the modal age of black bear dispersers support this hypothesis (e.g., Lindzey and Meslow 1977; Rogers 1987b). More importantly, from a genetic standpoint, delayed dispersal has the potential to result in the spatial association of kin males belonging to more than one generation, which would partly explain the patterns we observed.

Our results, however, are in strong contrast with data previously reported for black bears in New Mexico (Costello et al. 2008), which suggested that males in lower-density area dispersed less often or shorter distances than males in higher density areas. These authors argued that some males would respond to low density by remaining near their natal range, where competition from other males was lower than in higher density area. Higher densities and lower turnover of mature males (≥7 years old) would decrease the chances of mating for young males, probably making areas with low male density more appealing for establishment of a home range by a dispersing male (Costello et al. 2008 and references therein). However, Costello et al. (2008) also noticed that the estimated densities in their study area were overall relatively low even in higher density populations (≤17 bears/100 km^2^) (Costello et al. 2008) and well below the carrying capacity estimated in nearby populations. Such differences in population densities estimated in the study by Costello et al. (2008) in New Mexico and in the present study in Québec preclude any generalization on density-dependent dispersal behavior of black bears on its entire range, and points toward the importance of considering specific local conditions in interpreting determinism of dispersal.

In summary, and contrary to our initial hypothesis, our results suggest a negative density-dependent dispersal pattern in the American black bear in our study area, as previously reported in brown bear (i.e., Støen et al. 2006) as well as other mammals (e.g., Lambin 1994; Woodroffe et al. 1995; Ims and Hjermann 2001; Loe et al. 2009). Negative density-dependent dispersal has important implications for the evolution of dispersal in black bears. Restricted dispersal at higher densities would result in more opportunities for inbreeding and male-kin competition, since intermediate dispersal distances are normally required to avoid completely their occurrence (Ronce et al. 2001). We believe this could explain the moderate to high levels of genetic similarity observed among individuals in a high-density population of black bears reported in another part of the species range (Schenk and Kovacs 1996). However, inferences on the causes of dispersal become more complex when considering potential coevolution of kin recognition and dispersal as alternative ways to avoid inbreeding (Pusey and Wolf 1996; Perrin and Goudet 2001). Female mate choice before and even after mating (postcopulatory cryptic choice) was hypothesized in the Scandinavian brown bear population in which only ca. 2% of the litters resulted from the reproduction between fathers and daughters (Bellemain et al. 2006). Clearly, the question of potential female mate choice in black bears, as well as in other mammals, has to be investigated in future studies before any further inference can be made on the relative importance of its role in shaping patterns of dispersal.
